# The Laboratory of Genetics and Physiology 2: Emerging Insights into the Controversial Functions of This RIG-I-Like Receptor

**DOI:** 10.1155/2014/960190

**Published:** 2014-01-16

**Authors:** Zixiang Zhu, Xiangle Zhang, Guoqing Wang, Haixue Zheng

**Affiliations:** State Key Laboratory of Veterinary Etiological Biology, National Foot and Mouth Disease Reference Laboratory, Lanzhou Veterinary Research Institute, Chinese Academy of Agricultural Sciences, No. 1, Xujiaping Road, Lanzhou 730046, China

## Abstract

The laboratory of genetics and physiology 2 (LGP2) is a key component of the RNA helicase family of retinoic acid-inducible gene 1- (RIG-I-) like receptors (RLRs) and is widely involved in viral RNA recognition and regulation during innate immune responses. Unlike RIG-I and melanoma differentiation-associated 5, both RLR members, LGP2 lacks the caspase-recruitment domain (CARD), which is required for recruiting and interacting with downstream signaling proteins to activate a cascade of downstream signaling events. The absence of the CARD results in divergent functional performance for LGP2 compared to these other RLR members. Both negative and positive regulatory roles have been reported for LGP2 in antiviral immune responses. It is currently unclear how the unusual properties of LGP2 mediate opposing roles. Future studies should elucidate the molecular mechanism(s) of LGP2 action. This minireview provides a brief overview of LGP2 structure and functions, with an expanded discussion on the regulation mechanisms in response to viral infection, hopefully stimulating insight into the divergent roles of LGP2 in the regulation of antiviral immune responses.

## 1. Introduction

The secretion of type I interferons (IFNs) from virus-infected cells is part of a powerful and effective strategy allowing the host to launch both innate and adaptive immune responses and interfere with virus replication. In innate immunity, IFN production is triggered when host cells recognize molecules associated with pathogens. These pathogen-associated molecular patterns (PAMPs) are identified by cellular proteins with pattern recognition receptors (PRRs) [1, 2]. PRRs are directly responsible for sensing the presence of pathogens and activating innate immune responses and are also important for subsequent acquired immunity [3]. Currently, four major families of PRRs have been identified, including Toll-like receptors (TLRs), RNA helicase family of retinoic acid-inducible gene 1- (RIG-I-) like receptors (RLRs), NOD-like receptors (NLRs), and C-type lectin receptors (CLRs) [4].

The RLR family is one of the best characterized PRR families. IFN stimulation or virus infection can greatly enhance RLR expression. RLRs harbor two N-terminal caspase recruitment domains (CARDs): a central DEAD box helicase/ATPase domain and a C-terminal regulatory domain (RD). The RLR family is composed of RIG-I, melanoma differentiation-associated gene 5 (MDA5), and the laboratory of genetics and physiology 2 (LGP2) [5, 6]. The RLR members RIG-I and MDA5 contain two N-terminal CARDs; however, LGP2 does not [7]. The N-terminal CARD regions are responsible for recruiting and interacting with downstream signaling components, resulting subsequently in the transcriptional activation of various antiviral-related genes [8]. As LGP2 lacks the N-terminal CARD, LGP2 functions are expected to differ from RIG-I- and MDA5-mediated functions [9, 10].

Currently, the role of LGP2 in antiviral signaling is controversial [11, 12]. LGP2 has been previously reported to inhibit RIG-I signaling and activity both *in vivo* and *in vitro *[13, 14]. In contrast, MDA5-induced signaling transduction is stimulated in the presence of LGP2 [15]. In this review, we summarize the molecular properties of LGP2 and recent findings that implicate LGP2 in antiviral immunity, hopefully facilitating our understanding of how different studies lead to disparate conclusions about the biological activities of LGP2.

## 2. Structural and Biochemical Properties of LGP2

Even before the sequence analyses of the first cloned LGP2 were completed, it was apparent that LGP2 would be an unusual member of the RLR family. LGP2, also known as DHX58, was originally identified as a highly expressed gene in mammary tumors and is a cytoplasmic DEx(D/H)-box helicase that can recognize RNA [16]. LGP2 has significant sequence similarity with RIG-I and MDA5, with the exception of the CARD, which is required for the recruitment of the downstream interferon-beta promoter stimulator 1 (IPS-1) signaling protein. Thus, LGP2 lacks this signaling capacity.

**Figure 1:**
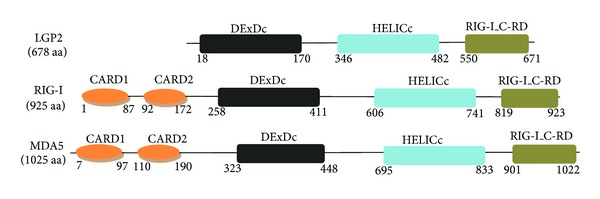
Structural representation of the RLRs. Key structural domains involved in RLRs signaling are shown. The RLRs consists of CARD: caspase-activation and recruitment domain; DExDc: DEAD-like helicase superfamily ATP binding domain; HELICc, helicase superfamily C-term domain associated with DExH/D box proteins; RIG-I_C-RD: C-terminal regulatory domain of RIG-I. LGP2 lacks the N-terminal CARDs.

The *LGP2* locus neighbors the *Stat3* and *Stat5* loci on mouse chromosome 11, as determined by the Laboratory of Genetics and Physiology [17, 18]. In humans, LGP2 is encoded by the *DHX58* gene, which is located on chromosome 17. The *DHX58* gene is 11,329 bp in length and contains 14 exons and 13 introns. The LGP2 protein is 678 amino acids with a molecular weight of approximately 75 kDa (Figure 1).

Although RIG-I and MDA5 have similar DExD/H-box helicase domains and CARDs, they recognize different viral RNA structures: RIG-I recognizes short (<1 kb) double-stranded RNAs (dsRNAs), free 5′ triphosphate end structures, and RNAs with complex secondary structures [19–21] whereas MDA5 detects long dsRNAs (>1 kb) and RNA molecules with “non-self-” 5′ termini [21–23]. In contrast, LGP2 RD binds to both dsRNA and single-stranded RNA (ssRNA) in a 5′-triphosphate-independent manner [6, 24]. LGP2 preferentially binds to a ligand containing a 5′-triphosphate, although it can bind RNA lacking a 5′-triphosphate [11]. In lysate, LGP2 can oligomerize in the presence of dsRNA, exhibiting a preference for binding to dsRNA rather than to ssRNA [11, 16]. The shapes of the LGP2 monomer in the absence of dsRNA and of the dimer complexed to a 27 bp dsRNA have been determined. The structures indicate a striking structural similarity to the helicase domain of the superfamily 2 DNA helicase, Hef [11]. The 2.0 Å resolution crystal structure of a human LGP2 CTD bound to an 8 bp dsRNA has also been reported, and it was indicated that LGP2 binds blunt-ended dsRNA of different lengths, composing complexes at a 2 : 1 stoichiometry [25].

### 2.1. The Negative Regulatory Roles of LGP2 in IFN Signaling

The role of LGP2 in antiviral immunity is not clear. Many studies have reported that LGP2 negatively regulates RLR signaling. Overexpression of LGP2 does not initiate the signaling transduction that leads to the activation of *IFN* transcription. In contrast, overexpression of LGP2 interferes with the sensing of viral RNA by RIG-I and MDA5 [13, 26]. Transfection of LGP2 and RIG-I at a 1 : 1 ratio into cells reduced RIG-I signaling by approximately half. At a 5 : 1 ratio, RIG-I activation of signaling was reduced to nearly background levels [11]. LGP2 expression is clearly induced by IFN-*β*, as are RIG-I and MDA5.

**Figure 2:**
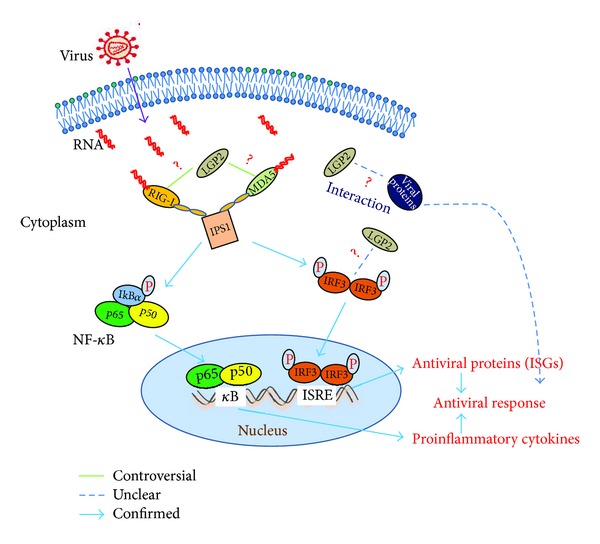
RLRs signaling pathway and the possible regulatory manners of LGP2. Viral dsRNA was recognized by RIG-I or MDA5, triggering signal transduction to nucleus and inducing the production of type I IFN and proinflammatory cytokines. The cooperative or competitive roles of LGP2 in RLR pathway activation are controversial. Whether the interaction between viral protein and LGP2 shows any antiviral response remains unclear, and the affection of LGP2 on IRF3 activation still needs more exploration.

Overexpression of LGP2 strongly inhibited virus-induced gene activation, as did a mutant of RIG-I lacking the CARD [26, 27]. In particular, overexpression of LGP2 inhibited the paramyxovirus Sendai (SV) and Newcastle disease virus (NDV) signaling to IFN-stimulated-regulatory-element- and nuclear-factor kappa-light-chain-enhancer-of-activated-B-cells- (NF-*κ*B-) dependent pathways. Interferon regulatory factor 3 (IRF3) activation was also reported to be reduced in LGP2 overexpressing cells infected with NDV [13], but the exact mechanism is still unclear (Figure 2). If LGP2 interacts with IRF3 and inhibits its phosphorylation should be studied in the future to understand this suppression action. In human cells infected with influenza A virus subtype H3N2 or other seasonal influenza A viruses (IAVs) that activate IRF3, overexpression of LGP2 or its repressor domain decreased *signal transducers and activators of transcription family 1* (*STAT1*) activation and *IFN* transcription approximately ten-fold. However, in both mouse and human cells infected with IAV subtype H1N1, which does not activate IRF3, LGP2 had no detectable role [28]. These studies imply that a potential negative-feedback regulatory role of LGP2 is possibly induced by decreasing IRF3 activation. RIG-I senses short dsRNA fragments produced from viral RNA with the RD region. Recognition activates IPS-1 and triggers IFN signaling pathways. However, LGP2 recognition of dsRNA does not induce IFN signaling pathways. In addition, the catalytically inactive helicase mutant of LGP2 is still capable of inhibiting RIG-I-induced IFN signaling pathways. Accordingly, it is proposed that LGP2 performs negative regulatory roles in antiviral signaling transduction process by sequestering dsRNA from RIG-I [26].

The presence of the RD in RIG-I impedes RIG-I-induced IFN signaling and deletion of the RIG-I RD resulted in constitutive signaling to the IFN-*β* promoter. RD expression alone prevented signaling and increased the cellular susceptibility to hepatitis C virus (HCV). An analogous RD in LGP2 interacts in *trans* with RIG-I to ablate self-association and signaling [16]. Moreover, it is reported that LGP2 expression inhibits SV- and dsRNA-initiated activation of the human *IFN-*β** gene promoter by preventing dsRNA and viral signal transduction to IRF3 and NF-*κ*B. In addition, downregulation of LGP2 expression levels enhanced *IFN-*β** mRNA expression as well as *RIG-I* and *MDA5* mRNA expression induced by SV. Correspondingly, upregulation of LGP2 decreased the SV-induced endogenous *IFN-*β**, *RIG-I*, and *MDA5* mRNA expression. This group also demonstrated that virus or dsRNA is independent of LGP2 to inhibit the RIG-I-induced antiviral signaling. LGP2 interacts with IPS-1 and competes with the kinase IKKi/IKK*ε* for the same interaction region on IPS-1, although it does not affect the interaction between RIG-I and IPS-1 [14]. The interaction between IPS-1 and IKKi/IKK*ε* is indispensable for IRF3 activation [29]; hence, this competition may explain the negative-feedback regulatory role of LGP2. Systematical analysis of the catalytic core with site-directed mutagenesis indicated that neither enzymatic activity nor RNA binding was required for negative regulation of antiviral signaling by LGP2. These results support an RNA-independent interference mechanism [30].

The V protein of paramyxoviruses can interact with LGP2 and the interaction cooperatively inhibited induction by RIG-I ligands, resulting in decreased RIG-I-dependent interferon induction [31]. But if this interaction will block virus particle assembly should also be considered (Figure 2). The R455 of LGP2 is required for recognition by measles V protein, but not for recognition by mumps virus, parainfluenza virus 5, or Nipah virus V proteins. These results indicate that paramyxoviruses have evolved distinct molecular interactions for LGP2 interference [32]. LGP2 knockout mice exhibited resistance to otherwise lethal infection with the negative-strand RNA vesicular stomatitis virus (VSV), supporting the hypothesis that LGP2 is a negative regulator for VSV [15]. However, LGP2 knockout mice exhibited defective type I IFN production in response to infection by the encephalomyocarditis virus (EMCV). These results taken together indicate a disparate regulatory role for LGP2 in the triggering of innate immune signaling pathways following RNA virus infection [15]. After these results came to light, the positive regulatory functions of LGP2 were reported.

### 2.2. The Positive Regulatory Roles of LGP2 in Antiviral Responses

In contrast to the reports of negative regulation by LGP2, targeted gene disruption studies have suggested a positive role for LGP2 in IFN-*β* induction and antiviral signaling transduction [33–36]. In addition, LGP2 differentially regulated RIG-I- and MDA5-dependent RNA sensing [24]. Although LGP2 has been shown to inhibit RIG-I signaling and activity, fully functional LGP2 was shown to be essential for the augmentation of MDA5-dependent signaling. LGP2 DExH domain or LGP2 RD alone was not sufficient for the synergistic effect on MDA5 activation, and more complex protein-protein or protein-ligand interactions might be involved in the mechanisms [24].

Studies of RLR signaling conducted by Satoh et al. provided evidence that LGP2 functions as a positive regulator during virus recognition and subsequent antiviral responses. This study found that viral dsRNA or 5′-triphosphorylated RNA can activate RIG-I or MDA5 by binding to their C-terminal domains (CTDs), and LGP2 facilitated RIG-I and MDA5 recognition of viral RNA. LGP2 knockout mice (LGP2^−/−^) and LGP2^K30A/K30A^ mice, in which the helicase domain was mutated with a lysine-to-alanine substitution at position 30 to abolish ATPase activity, were generated. The experimental data suggested that both LGP2^−/−^ and the LGP2^K30A/K30A^ mice were highly susceptible to EMCV infection and that the activation of NF-*κ*B and IFN-stimulated regulatory elements in response to EMCV infection were severely attenuated in LGP2^−/−^ cells. In addition, phosphorylation of STAT1 was found to be abrogated in LGP2^−/−^ cells. Overexpression of LGP2 in LGP2^−/−^ cells could restore the virus-mediated IFN responses. Although LGP2 and its ATPase activity were previously reported to be dispensable for the responses to synthetic RNA ligands for MDA5 and RIG-I, this study suggested that LGP2 facilitated viral RNA recognition by RIG-I and MDA5 through its ATPase domain [35]. Chang et al. determined MDA5 and LGP2 homologues of rainbow trout (*Oncorhynchus mykiss*) and found an additional LGP2 variant with partial CTD of RIG-I. Treatment with polyinosinic-polycytidylic acid (poly(I:C)), recombinant IFN, or RNA virus infection up-regulated trout LGP2 expression remarkably. Overexpression of LGP2 and MDA5, but not of the LGP2 variant, significantly enhanced the expression of *Mx* transcripts in transfected cells, and this enhancement clearly correlated with the inhibition of virus replication. These results suggest that both MDA5 and LGP2 are important RLRs in host immune responses against infection of RNA viruses. LGP2 variant with a deletion of 54 amino acids at the CTD performed negative roles in LGP2-elicited antiviral reactions by competing for the viral RNA PAMPs [37]. Some antibodies against human LGP2 can detect two bands on western blot (approximately 75 and 23 or 45 kDa) (Anti-DHX58 antibody, Abcam), and some additional bands can also be observed when detecting LGP2 in mouse tissues with antibodies against mouse LGP2 by western blot (approximately 26, 36, 48, and 55 kDa) (Anti-DHX58 antibody, abcam). Whether these extra bands are the variants or isoforms of LGP2 in these mammalian cells remains unclear. The roles and identities of these proteins should be determined in future studies. Possibly, the different performance of LGP2 can be understood based on that.

LGP2 was further found to participate in cellular responses to cytosolic double-stranded DNA (dsDNA) [34]. Although our understanding of cellular responses to pathogens with RNA genomes by way of the RLR pathways is rapidly growing, the responses to pathogens with DNA genomes remain unclear [34, 38, 39]. In LGP2-deficient mice, *Listeria monocytogenes* infection impaired secretion of type I IFN and clearly promoted bacteria replication [34]. Similar results were observed with vaccinia virus infection, confirming positive regulation of LGP2 in antiviral signaling pathways. These mechanistic studies determined that LGP2 did not bind directly to DNA but instead mediated the antiviral responses indirectly. Thus, LGP2 might be acting downstream of the intracellular RNA polymerase III pathway to trigger antimicrobial signaling [34]. It was deduced that LGP2 may positively regulate antiviral responses by functioning upstream of RIG-I and MDA5 and its adaptor, IPS-1. Optimal cytokine responses mediated by the MDA5-IPS-1 pathway and TLR-mediated sensing are essential for antiviral activity against modified vaccinia virus Ankara (MVA) infection. siRNA knockdown of MDA5 and IPS-1, but not of RIG-I, led to the impairment of a cascade of IFN responses to MVA infection [40, 41]. Similar siRNA knockdown studies suggested that IFN responses to poly(dA-dT) were principally dependent on RIG-I but not on MDA5 [40, 42–44]. In LGP2-deficient cells, MVA infection severely impaired cytokine production. poly(dA-dT) signaling was also impaired in LGP2-deficient cells. Based on these observations, it was hypothesized that LGP2 positively contributes to both RIG-I- and MDA5-mediated signaling [34].

Transcriptional activity of the LGP2 promoter and LGP2 expression were shown to be strongly enhanced by IRF3 in olive flounder (*Paralichthys olivaceus*) after viral infection or stimulation with poly(I:C): this result implies a crucial role for LGP2 in RLR signaling [45]. Liniger et al. identified chLGP2 in chickens and proved that it functioned as a positive regulator of chMDA5 signaling. Although overexpression of LGP2 inhibited chIFN-*β* promoter activation, silencing of endogenous chLGP2 was reported to dramatically reduce chIFN-*β* expression induced by IAV [46]. A recent study demonstrated that ATP hydrolysis promotes RNA recognition and antiviral immune responses with the innate immune sensor LGP2. Basal ATP hydrolysis, but not dsRNA-stimulated ATP hydrolysis, by LGP2 was required for enhanced dsRNA recognition. LGP2 ATP hydrolysis was indispensable for enhanced MDA5-mediated IFN signaling [7], confirming the positive regulatory effect of LGP2 on IFN signaling. Previous publications reported that LGP2 impairs IFN production by poly(I:C) when both LGP2 and poly(I:C) are at high levels [14]. A recent study showed that LGP2 acts as a potent stimulator of poly(I:C) signaling when limited poly(I:C) are transfected in cells, and, thus, the expression level of LGP2 is critical for determining cellular sensitivity to initiation. The expression level of LGP2 may directly determine whether the regulatory role of LGP2 in the overall IFN signaling to dsRNA is positive or negative [47].

## 3. Summary

LGP2, an important member of the RLR family, has been found to be heavily involved in virus-triggered immune responses [48, 49]. The absence of the CARD in the *LGP2* gene sequence results in function that is different from two other RLR family members: RIG-I and MDA5, both of which contain the CARD (Table 1) [11, 18]. At present, the interaction and sensing of LGP2 with its corresponding ligand RNA is less understood and studied than ligand-receptor interaction of RIG-I or MDA5.

Mouse fibroblast cells were widely used in LGP2 antiviral activity study; it was found that the roles of LGP2 in IAV-infected chicken macrophage cells, chicken embryonic cells, and human epithelial cells reflected similar activity compared with its activity in mouse cells. It seems that the contradictory regulatory roles of LGP2 may not correlate with the cell-type or species difference [28, 50]. Is the opposing responses associate with the structure of the virus? The exact possibility has not yet been confirmed and more studies should explore this in the future. Although the structural similarity to RIG-I and MDA5 suggested that LGP2 binds dsRNA [51, 52], the exact roles and mechanisms of LGP2 in RNA recognition and antiviral signaling remain unclear and controversial. This lack of clarity is due, in part, to contradictory LGP2 functions reported for different experimental approaches or methods. Different types of virus may trigger different functional performance of LGP2. RIG-I and MDA5 bind to different ligands, but both of them finally induce the same antiviral activity, inducing the production of type I IFN and proinflammatory cytokines in a precisely regulated and balanced manner [53, 54]. In this process, LGP2 exhibits either negative or positive regulation of RIG-I- and MDA5-mediated antiviral signaling and, accordingly, has been characterized as both an activator and a feedback inhibitor of RLR signaling. It is possible that LGP2 is a balancer to regulate proper signaling transduction triggered by RIG-I and MDA5.

A recent study reported that LGP2 was not indispensable for the activation of innate immune responses against viral infection but was essential for controlling antigen-specific CD8(+) T-cell survival and fitness during peripheral T-cell-number expansion against viral infection [55]. LGP2 promoted an essential prosurvival signal in response to antigen stimulation to confer CD8(+) T-cell-number expansion and effector functions against West Nile virus and lymphocytic choriomeningitis virus; CD95 was involved in this signaling transduction [55]. Although the MDA5/LGP2 interaction was excluded to perform this regulatory function, whether RIG-I referred to this process remained unclear. In conclusion, these results indicate that LGP2 promotes antiviral immune responses through the cell-intrinsic regulation of CD8+ T-cell survival and effector function [56]. Future studies that focus on understanding the role of LGP2 in cellular immunology are necessary to improve our understanding of the precise functions of LGP2 in antiviral immune responses.

## Figures and Tables

**Table 1:** RLR ligands and viruses reported to associate with each RLR.

Viral nucleic acid features (PAMPs)		ATP binding sites	Role in antiviral immune response	Viruses
LGP2	dsRNA, ssRNA, and free 5′-triphosphate end structures	446, 467, 471, 473	Positive	Encephalomyocarditis, vaccinia,and mengo
			Negative	Paramyxovirus Sendai, vesicular stomatitis, Newcastle disease, and influenza A
RIG-I	Short dsRNAs (<1 kb), free 5′-triphosphate end structures, and complex secondary RNA structures	271~276	Positive	Reovirus, dengue, West Nile, rotavirus, Sendai, Vesicular stomatitis, respiratory syncytial, measles, rabies; influenza A, influenza B, ebola, hepatitis C, Japanese encephalitis, and Newcastle disease
MDA-5	Long dsRNAs (>1 kb), ‘‘non-self-” RNA 5′-termini	332~426	Positive	Reovirus, dengue, West Nile, rotavirus, Sendai, encephalomyocarditis, mengo, Theiler, polio, and murine norovirus
